# Supplementary material for: Identifying transgene insertions in Caenorhabditis elegans genomes with Oxford Nanopore sequencing
Source: PeerJ. 2024 Sep 13;12:e18100. doi: 10.7717/peerj.18100 (PMC11404476; doi:10.7717/peerj.18100)
Supplement: Supplemental Information 1 — Canu-corrected reads were used to assemble the genomes with Flye. [file peerj-12-18100-s001.pdf]

**Table S1.** Sequencing statistics for UA44 and BY250 before and after Canu-Correction. Canu-corrected reads were used to assemble the genomes with Flye.

**Additional Sequencing Statistics**

| | Total Bases (Gb) | Number of Reads | Mean Read Length | Median Read Length | N50 | Coverage |
|---|---|---|---|---|---|---|
| UA44 | 7.44 | 2,673,711.0 | 2,783.80 | 1,564.00 | 4,732.0 | 74.22 |
| Corrected | 3.88 | 597,106.00 | 6,498.10 | 4,190.0 | 8,082.0 | 38.69 |
| BY250 | 12.45 | 4,054,501.0 | 3,070.00 | 1,540.00 | 6,116.0 | 124.12 |
| Corrected | 4.10 | 344,582.0 | 11885.10 | 10,929.0 | 15109.00 | 40.84 |
