# Supplementary material for: Identifying transgene insertions in Caenorhabditis elegans genomes with Oxford Nanopore sequencing
Source: PeerJ. 2024 Sep 13;12:e18100. doi: 10.7717/peerj.18100 (PMC11404476; doi:10.7717/peerj.18100)
Supplement: Supplemental Information 2 — All partial hits to UA44 did not include the a-synuclein gene. [file peerj-12-18100-s002.pdf]

Table S2. Partial vector hit location information for UA44 and BY250. All partial hits to UA44 did not include the α-synuclein gene.

Partial Potential Insertion Locations

Line	Sequence		Location		Insertion			Blast Score Information					
	ID	Length	Start	Stop	Start	Stop	Length Inserted	Match %	Matches	Length	Gaps	Bit Score	Evalue
UA44	IV	17.96 Mb	8782175	8784655	2439	4924	2485	99%	2480	2487	7	4547	0
			8779577	8780298	18	739	721	99%	721	722	0	1328	0
			(-) 8762877	8762158	18	739	721	99%	718	722	2	1310	0
			8781454	8782175	1239	1966	727	99%	721	728	6	1301	0
			(-) 8761004	8760434	1239	1808	569	97%	560	578	15	953	0
			(-) 8778290	8777842	4466	4924	458	98%	448	459	10	778	0
			8759283	8759579	4427	4747	320	98%	316	321	5	560	8.00E-158
			(-) 8759770	8759589	4545	4729	184	96%	179	186	5	300	2.00E-79
			8778733	8779300	18	662	644	76%	493	652	91	239	5.00E-61
			13698698	13698890	1888	2078	190	83%	164	197	10	172	5.00E-41
	I	15.37 Mb	(-) 15154594	15153371	1207	2432	1225	99%	1222	1227	4	2235	0
			(-) 15229897	15229764	2226	2360	134	82%	111	135		115	8.00E-24
			(-) 3094739	3094677	2375	2432	57	86%	54	63	5	62.1	1.00E-07
	III	14.02 Mb	9406356	9407072	23	739	716	100%	717	717	0	1325	0
	V	21.24 Mb	20767099	20767210	2330	2429	99	81%	91	112	12	80.5	3.00E-13
BY250	I	15.37 Mb	(-) 11059035	11058951	48	132	84	100%	85	85	0	158	3.00E-37
