# Supplementary material for: Identifying transgene insertions in Caenorhabditis elegans genomes with Oxford Nanopore sequencing
Source: PeerJ. 2024 Sep 13;12:e18100. doi: 10.7717/peerj.18100 (PMC11404476; doi:10.7717/peerj.18100)

Figure S2. Syntenic Information for BY250 genome after scaffolding. A) shows the chromosome alignment for BY250 and N2; B) shows the syntenic map of BY250 and N2; and C) shows the syntenic depth or ratio of BY250 genes to N2 genes.

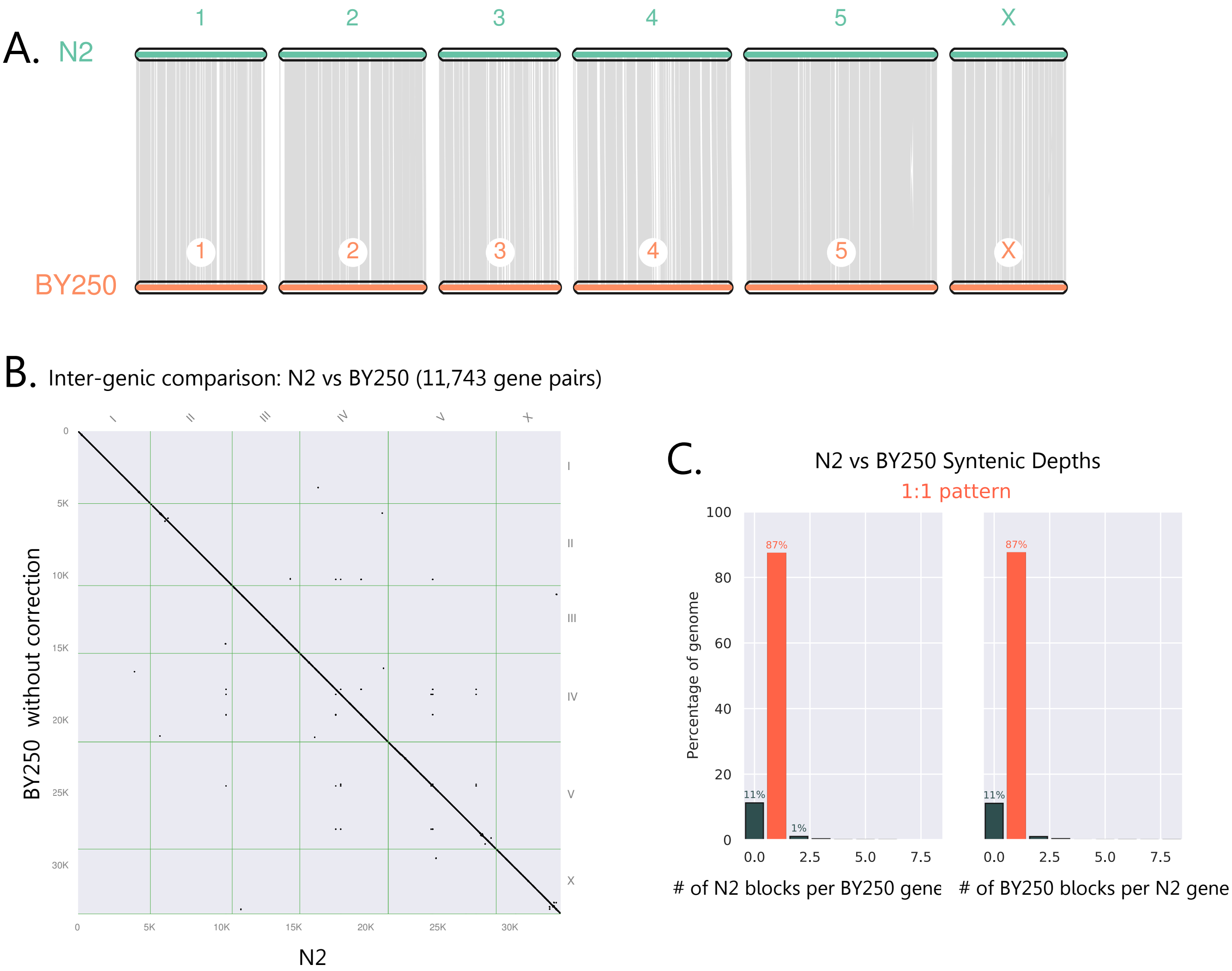

Supplement: Supplemental Information 4 [file peerj-12-18100-s004.pdf]
